# Subtle Variations in Surface Properties of Black Silicon Surfaces Influence the Degree of Bactericidal Efficiency

**DOI:** 10.1007/s40820-017-0186-9

**Published:** 2018-02-06

**Authors:** Chris M. Bhadra, Marco Werner, Vladimir A. Baulin, Vi Khanh Truong, Mohammad Al Kobaisi, Song Ha Nguyen, Armandas Balcytis, Saulius Juodkazis, James Y. Wang, David E. Mainwaring, Russell J. Crawford, Elena P. Ivanova

**Affiliations:** 1 School of Science, Faculty of Science, Engineering and Technology, Swinburne University of Technology, Hawthorn, VIC 3122 Australia; 2 Departament d’Enginyeria Química, Universitat Rovira i Virgili, Avinguda dels Països Catalans 26, 43007 Tarragona, Spain; 3 Melbourne Center for Nanofabrication, 151 Wellington Road, Clayton, VIC 3168 Australia; 4 School of Science, RMIT University, Melbourne, VIC 3001 Australia

**Keywords:** Black silicon, Nanoarchitecture, Bactericidal efficiency, Deep reactive ion etching (DRIE), Neural network analysis

## Abstract

One of the major challenges faced by the biomedical industry is the development of robust synthetic surfaces that can resist bacterial colonization. Much inspiration has been drawn recently from naturally occurring mechano-bactericidal surfaces such as the wings of cicada (*Psaltoda claripennis*) and dragonfly (*Diplacodes bipunctata*) species in fabricating their synthetic analogs. However, the bactericidal activity of nanostructured surfaces is observed in a particular range of parameters reflecting the geometry of nanostructures and surface wettability. Here, several of the nanometer-scale characteristics of black silicon (bSi) surfaces including the density and height of the nanopillars that have the potential to influence the bactericidal efficiency of these nanostructured surfaces have been investigated. The results provide important evidence that minor variations in the nanoarchitecture of substrata can substantially alter their performance as bactericidal surfaces.

**Electronic supplementary material:**

The online version of this article (10.1007/s40820-017-0186-9) contains supplementary material, which is available to authorized users.

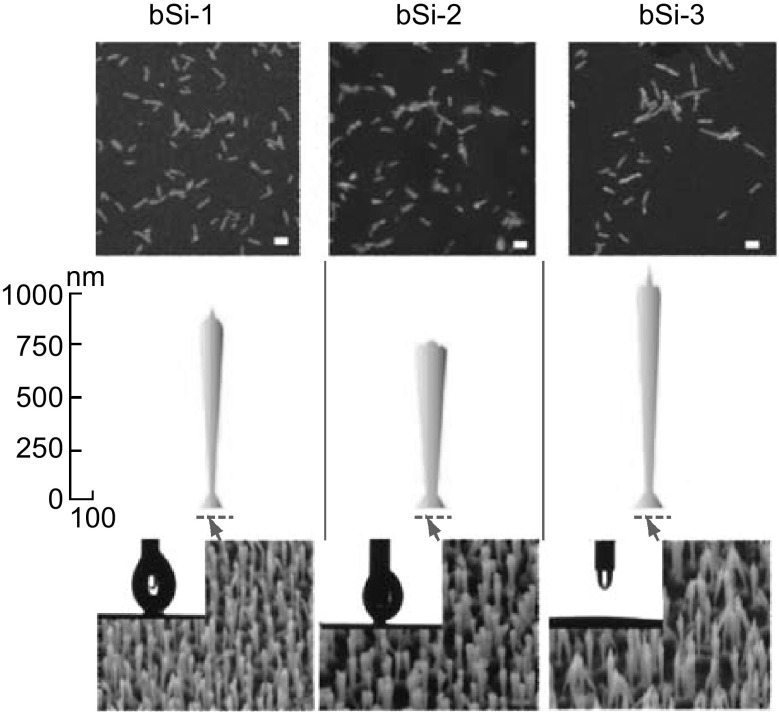

## Highlights

- Three types of black silicon (bSi) surface were successfully fabricated using deep reactive ion etching with pillar heights (652.7–1063.2 nm) and density (8–11 tips per µm^2^).
- Less bactericidal bSi surfaces were found to contain nanopillars of heights reaching 1000 nm that were not always well separated, lower pillar density (8 tips per µm^2^), and aspect ratios of 8.8.

## Introduction

The recent discovery that some nanostructured surfaces exhibit a mechano-bactericidal effect [1–6] has prompted a number of extensive studies to be undertaken toward the fabrication of new nanopatterned materials that also possess antibacterial properties [7–14]. Much of this inspiration has been drawn from naturally occurring bactericidal surfaces such as those possessed by cicada *Psaltoda claripennis* and dragonfly *Diplacodes bipunctata* wings [1, 5]. It has been noted in some studies that the efficiency with which the cells rupture by nanostructures may be dependent on the nanopattern of the surface [1–3, 15, 16], e.g., defined surface parameters of 280-nm pillars height, approximately 60-nm tip diameter and approximately 60-nm spacing have proven to be more effective at killing 85% and 89% of *Staphylococcus aureus* and *Pseudomonas aeruginosa*, respectively [13]. It was also reported that approximately 99% of the *Pseudomonas aeruginosa* cells coming into contact with the surface of *Psaltoda claripennis* wings have been shown to be eliminated [4]. Kelleher et al. [3] reported that the pillars present on the wings of some cicada species, with average height dimensions of 180–250 nm, were able to eliminate approximately 100% of *Pseudomonas fluorescens* cells coming into contact with the surface.

The black silicon (bSi) surface is the first synthetic analog of natural bactericidal surfaces of the *Diplacodes bipunctata* dragonfly wing [1]. Previously bSi surfaces have been widely used in renewable energy applications such as photovoltaic and solar cells due to their property of having the low reflectivity [17, 18] and were successfully fabricated using a number of nanofabrication techniques including reactive ion etching [19], electrochemical etching [20], and laser treatment [21]. Biomimetic bSi was demonstrated to be effective toward different types of bacteria, including common human pathogens such as *Pseudomonas aeruginosa* and *Staphylococcus aureus* [1].

Despite the striking similarity between the natural and synthetic nanostructured surfaces, some variations in bactericidal activity were observed among wings of different dragonfly species [2] and the biomimetic bSi surfaces [1]. In light of these results, the aim of the current work was to investigate the relationship between the characteristics (nanopillar density, height, and interpillar distance) of the bSi nanopatterned surfaces and their bactericidal efficiencies. These data provide useful insights into way in which the design and fabrication of mechano-responsive antibacterial surfaces can be made more effective.

## Materials and Methods

### Reactive Ion Beam Etching

Reactive ion etching (RIE) using SF_6_ and O_2_ was performed for 5 min to produce the nanopillars on the surface of silicon wafers (WRS Wafers) using an Oxford PlasmaLab 100 ICP380 instrument. The nanopillars were fabricated with a high degree of precision in terms of accuracy in size and position, allowing for a systematic study of the surface topology [1, 19]. Details of reactive ion etching are found in Supplementary Data Section S1.1. Three fabricated bSi surfaces, designated bSi-1, bSi-2, and bSi-3, were investigated in this study for their surface characteristics and bactericidal efficiency.

### Contact Angle Measurements

Static water contact angles were measured on the bSi surfaces using the sessile drop method [22, 23]. The contact angle measurements were carried out in air using an FTA1000c instrument equipped with a nanodispenser (First Ten Ångstroms, Inc., Portsmouth, VA, USA.). The volume of the droplets used for analysis was approximately 1.0 µL. The contact angles were measured by recording 50 images over 2 s with a Pelcomodel PCHM 575-4 camera and measuring the contact angles after the droplet had been rested on the surface for approximately 1 s. The surface wettability was determined on five different locations on the surface of three separate bSi samples of each type of the surfaces.

### XPS Analysis

X-ray photoelectron spectroscopy (XPS) was performed using a Kratos Axis Ultra DLD X-ray photoelectron spectrometer (Kratos Analytical Ltd., UK) equipped with a monochromatic X-ray source (Al Kα, *hυ* = 1486.6 eV). Details of reactive ion etching are found in Supplementary Data Section S1.2. The relative atomic concentration of the elements detected by XPS was quantified on the basis of the peak area in the survey spectra, using the sensitivity factors appropriate for the Kratos instrument. High-resolution scans were performed across each of the C 1s, O 1s, F 1s, and Si 2s peaks.

### Surface Characterization

The surface topography and architecture were analyzed using an Innova scanning probe microscope (Bruker, USA). Scans were performed in tapping mode under ambient temperature and pressure conditions, using silicon cantilevers (MPP-31120-10, Veeco, USA.) with a spring constant of 0.9 N m^−1^ and a resonance frequency of approximately 20 kHz. Scanning was performed perpendicular to the axis of the cantilever at a scan speed of 1 Hz. Initially, 2.5 × 2.5 µm^2^ areas were analyzed to evaluate the overall homogeneity of the surface, prior to generating topographical profiles at five different locations on each bSi surface.

High-resolution electron micrographs of the bSi surfaces were recorded using a field-emission scanning electron microscope (FE-SEM; ZEISS SUPRA 40 VP, Oberkochen, BW, Germany) at 3 kV under 10,000×, 30,000×, 70,000×, and 110,000× magnification using the method described in our previously published studies [1, 2]. The nanopillared patterns present on the bSi surfaces were analyzed using ImageJ^®^ software package using a fast Fourier transform (FFT) algorithm [24, 25]. The average FFT images are obtained by averaging over FFT transformed tiles of 512 × 512 pixels fitting into SEM images at 10,000× magnification with displacements of 100 pixels to each other.

### Neural Network Analysis for Pillar Tip Detection and Distribution

**Fig. 1:**
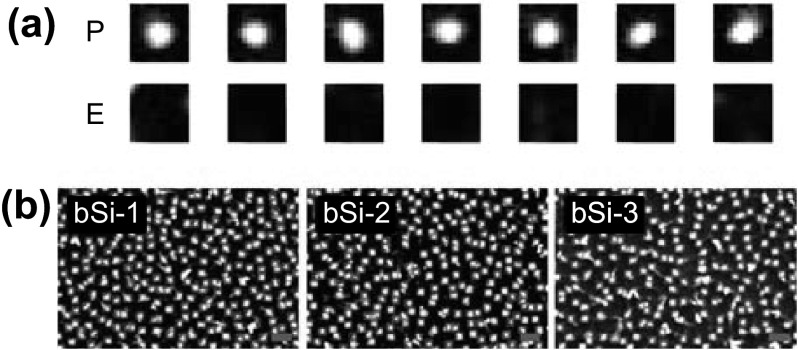
Identification and detection of the nanopillars of the black silicon surfaces. **a** Training set based on the SEM images of bSi-1 (×10,000 magnification) used to distinguish pillar tips and free regions between the pillars. P: pillar tip; E: empty space between pillars. **b** Detected pillar tips (red squares) on each type of bSi surface. Scale bars correspond to 500 nm. (Color figure online)

A fully connected, three-layer back-propagation neuronal network, as described in Zheng et al. [26] was employed in this study for the detection of the pillar distribution in SEM images. The input layer of the network was prepared to receive 12 × 12 pixel images, with three color channels totaling up to 12 × 12 × 3 input neurons. In the case of black and white SEM images, three input neurons per pixel with the same input value were used. A total of 24 neurons in the hidden layer were utilized. The output layer consists of two neurons corresponding to the two classes E (empty space between pillars) and P (pillar tip) with respect to which each pixel and its neighborhood were classified. A training set of 14 images was created, based on sample bSi-1 focusing on seven positions of pillars (species P) and seven positions between pillars (species E) (Fig. 1a).

An extended training set of 504 pictures per species was generated by rotating each of the seven pictures by 24 equidistant angles, as well as rescaling to 90, 100, and 110% of the original size. Training was performed by (on average) 2000 forward passes per extended training set picture, with full error back propagations. Feed-forward and error-backward propagations employ well-known sigmoidal characteristics based on the logistic function. The target signals for the two output neurons were [1, 0] corresponding to E, and [0, 1] corresponding to P, respectively. The pillar tip positions in the SEM micrographs were detected by forward passing patches of 12 × 12 pixels through the network. Pillar tip positions have been identified by finding the regions of maximum response of the second output neuron (Fig. 1b).

The probability distribution for finding neighboring pillars as a function of distance by numerical estimation of the pair-correlation function was calculated according to:

1 $$g\left( R \right) \, = \, \rho \left( R \right)/\rho_{0}$$

where *ρ*(*R*) is the average pillar tip number density as a function of the distance *R*, between pillar tips, with *ρ*_0_ normalizing *g*(*R*) to a value of 1 for large distances.

### Bacterial Strains, Growth Conditions and Sample Preparation

Two strains of pathogenic bacteria, *Pseudomonas aeruginosa* ATCC 9027, *Staphylococcus aureus* CIP 65.8^T^, which are responsible for a large number of postoperative infections, were obtained from the American Type Culture Collection (ATCC, Manassas, VA, USA) and the Culture Collection of the Institute Pasteur (CIP, Paris, France) [1, 27]. The selected strains are representatives of two large prokaryotic lineages, namely Gram-negative and Gram-positive bacteria, whose responses on nanostructured surfaces will be typical for taxonomically related bacterial taxa. Prior to each experiment, bacterial cultures were refreshed on nutrient agar from stocks (BD, USA). Fresh bacterial suspensions were grown overnight at 37 °C in 5 mL of nutrient broth (BD, USA). Bacterial cells were collected at the logarithmic stage of growth (data not shown), and the density of bacterial suspensions was adjusted to OD_600_ = 0.1.

### Cell Viability Analysis

Confocal laser scanning microscopy (CLSM) was used to visualize the proportions of live and dead cells using a LIVE/DEAD^®^ BacLight™ Bacterial Viability Kit, L7012. A mixture of SYTO^®^ 9 and propidium iodide fluorescent dyes (Molecular Probes™, Invitrogen, Grand Island, NY, USA). SYTO^®^ 9 permeated both intact and damaged membranes of the cells, binding to nucleic acids and fluorescing green when excited by a 485-nm wavelength laser. Propidium iodide alone entered only the cells with significant levels of membrane damage, which are considered to be non-viable, and binds with higher affinity to nucleic acids than SYTO^®^ 9. Bacterial suspensions were stained according to the manufacturer’s protocol and as described in our previously published articles [1, 5, 28] and imaged using a FluoView FV10i inverted microscope (Olympus, Tokyo, Japan).

To quantify the bactericidal efficiency of bSi surfaces, two bacterial strains were incubated on each type of the surfaces for 3 h. Viability assays of *P. aeruginosa* and *S. aureus* cells were performed using a standard plate count assay [29]. Further details are given in Supplementary Data Section S1.3. The number of colony-forming units was regarded as being equivalent to the number of live cells in suspension [29]. The bactericidal efficiency was estimated as the number of inactivated cells per square centimeter of surface, per minute of incubation time, relative to the control surfaces.

### Statistical Analysis

Data were expressed as mean ± standard error of the mean. For comparison of two groups, *p* values were calculated by two-tailed paired Student’s *t* test. In all cases, *p* values < 0.05 was considered to be statistically insignificant.

## Results and Discussion

### Characterization of Black Silicon Surfaces

Three types of bSi surfaces, fabricated using RIE, were investigated in this study. It was found that some variation in the nanopillar pattern reflected in differences of pillar heights, aggregation, and surface wettability (Table S1 and Fig. S1A). Water contact angle (WCA) measurements indicated that while the bSi-1 and bSi-2 surfaces were found to be highly hydrophobic with a WCA of 130° and 100°, respectively, bSi-3 surfaces were the highly hydrophilic (Table 1). An XPS analysis of the bSi samples revealed some variations in the silicon ratio in both its elemental Si and oxidized SiO_2_ states (Table S1 and Fig. S1A). These variations might be a contributing factor toward surface hydrophilicity (Fig. S1A). This change in surface hydrophilicity was most likely attributable to the increased proportion of oxidized silicon in comparison with the non-structured Si surface. Since previously published works provided evidence that the surface nanopattern morphology and not chemical composition and/or wettability play a role in mechano-bactericidal activity [1, 5, 13], the bactericidal activity of studied bSi substrata was compared and contrasted only in the context of surface nanopattern parameters.

**Table 1:** Summary of surface wettability, surface roughness analysis and geometrical parameters of nanopillars of the bSi surfaces

Parameters	bSi-1	bSi-2	bSi-3
Wettability			
Water contact angle, *θ* (degrees)	130.8 ± 3.2	100.9 ± 1.6	8.1 ± 1.2
Surface roughness^a^			
Average roughness (nm) *R*_a_	82.3 ± 29.6	110.3 ± 27.6	124.7 ± 17.7
Root-mean-square roughness (nm) *R*_q_	103.7 ± 37.3	136.5 ± 34.2	156.8 ± 22.2
Geometrical parameters^b^			
Height (nm)	836.8 ± 91.2	657.9 ± 74.3	1063.2 ± 159.5
Tip width (nm)	100.1 ± 36	110.3 ± 26.9	120.5 ± 17.1
Interpillar distance (nm)	153.1 ± 55.3	135.6 ± 33.9	197.4 ± 28.0
Density (number of tips per µm^2^)	11 ± 4	10 ± 3	8 ± 2
Aspect ratio	8.4 ± 2.9	6.0 ± 1.8	8.8 ± 2.0
Chemical composition^c^			
Si/SiO_2_	1.2	1.3	2.2
Si (total) (At.%)	26	27	35

^a^AFM roughness analysis, 2.5 × 2.5 µm^2^ scanning areas

^b^SEM micrographs

^c^XPS analysis

**Fig. 2:**
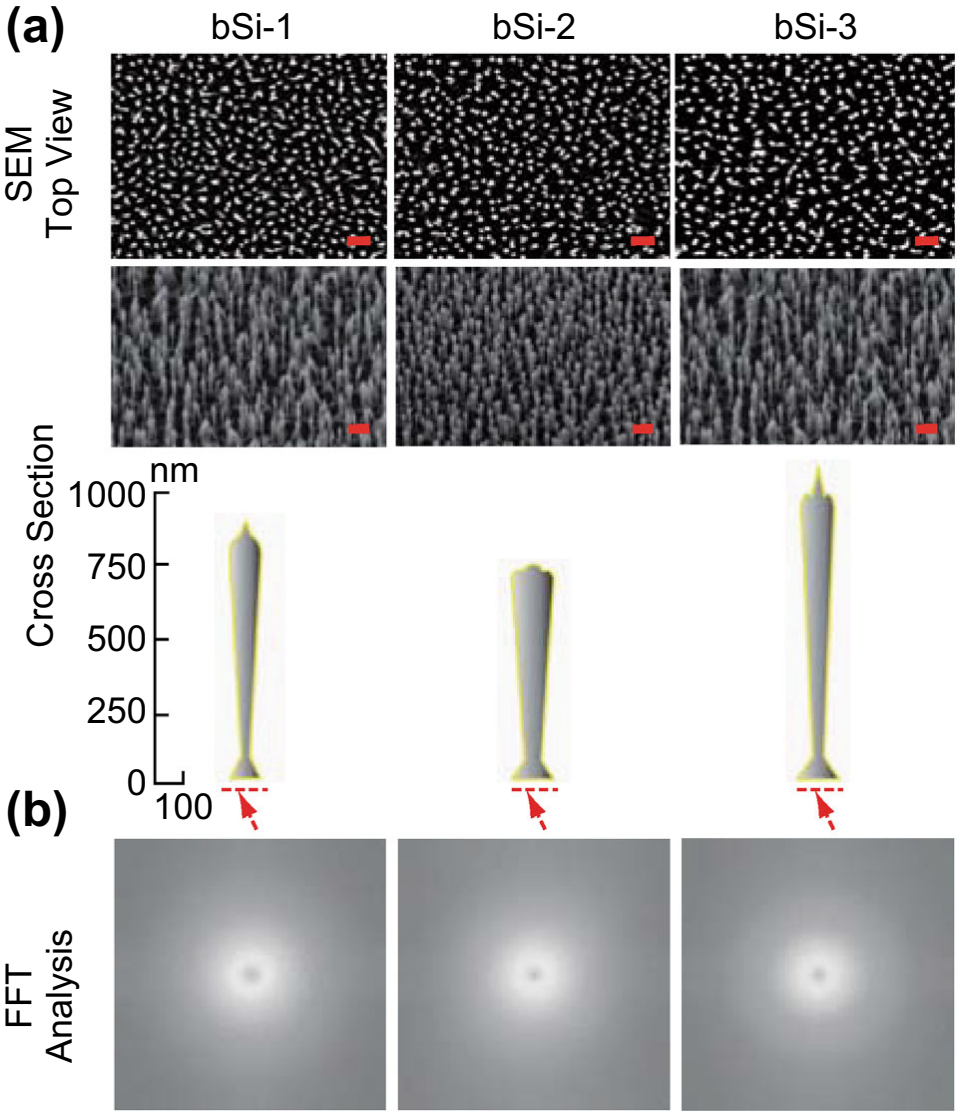
Comparative analysis of the bSi surface nanoarchitecture. **a** The upper plane of the surfaces on the SEM images, ×20,000 magnifications, and scale bar is 1 µm. The distinct morphologies of the nanopillars present on each type of bSi surface as seen from the SEM micrographs taken at a tilted angle of 45° (SEM cross sections) from the baseline, ×30,000, scale bar is 200 nm. The inset shows a schematic depiction of the representative shapes of the nanopillars present on bSi surfaces derived from side-view SEM images, highlighting the distinct pillar morphology including the pillar height and tip width. **b** The average fast Fourier transform of tiles of size 512 × 512 pixels for each of the species. The center pixel has been replaced by the averaged gray value

Examination of the optical, AFM, and SEM images of the bSi surface nanopatterns underlined the random distribution of the nanopillars on all bSi surface types (Fig. 2a and Figs. S2–S3). The surface roughness, geometrical parameters, and detailed schematic analysis of the shape of the nanopillars are presented in Table 1 and Fig. 2b. The geometry of the individual pillars showed a reduced cross-section diameter when moving to the base, where sometimes the tapering at the waist would reduce the mechanical resistance of the pillars to a level such that the pillars were able to touch reach each other at their top due to capillary forces (Fig. 2b). The bSi-1 surface was found to possess well-separated pillars, which were approximately 625 nm long and 81 nm in diameter at their widest cross section close to the tip. These pillars dimensions were found to be similar to those present on the surface of the bSi-2 samples. The nanopillars present on the bSi-3 surface were found to be taller, with an average height of approximately 1063 nm, and wider at their tips (120 nm) and sharply tapered (Fig. 2b, Fig. S2). The aspect ratio of the bSi surfaces ranged between 6.0 and 8.8.

The bearing ratio of each bSi surface type was determined from the statistical AFM data, since the protrusion curvatures were present in both the *x*, *y*, and *z* (height) planes. The variation in the bearing ratio was dependent on the height of the nanopillars, as shown in Figs. S3 and S4. The bearing curve reflected the surface area at a specific depth with respect to the entire area being analyzed. For all samples shown in Fig. S4, at a height of about 200 nm, the bearing curve started to increase in the bearing area fraction and approaches a constant bearing area level within several 100 nm. The increase indicated the volume of everything present above the surface, which is the volume of the pillars. It appeared that the bSi-1 and bSi-2 samples reached a constant bearing area level at approximately 500 nm, while the bSi-3 samples reached a constant bearing area level at approximately 800 nm, highlighting the nanopillar height present on each surface.

A FFT analysis of the top view of the SEM images of the bSi surfaces confirmed the isotropy of the nanopillar pattern, showing a variation in the average distance between the pillars resulting in a broad halo ring in the FFT images (Fig. 2b and Fig. S1B). The halo ring peak position is found in a range of wave numbers (*q*) between 0.016 and 0.021 (nm^–1^) indicating a most pronounced regularity of surface structures on scale of approximately *R* = 330 and 390 nm. The radially averaged Fourier spectra are shown in Fig. S1B for all samples bSi-1 to bSi-3. No significant differences between the radial spectra of the samples bSi-1 to bSi-3 were detected. Note that the shown spectra convolute spatial relations between pillars as well as the SEM projected internal pillar structures.

### Neural Network Analysis and Pillar Pair-Distributions

**Fig. 3:**
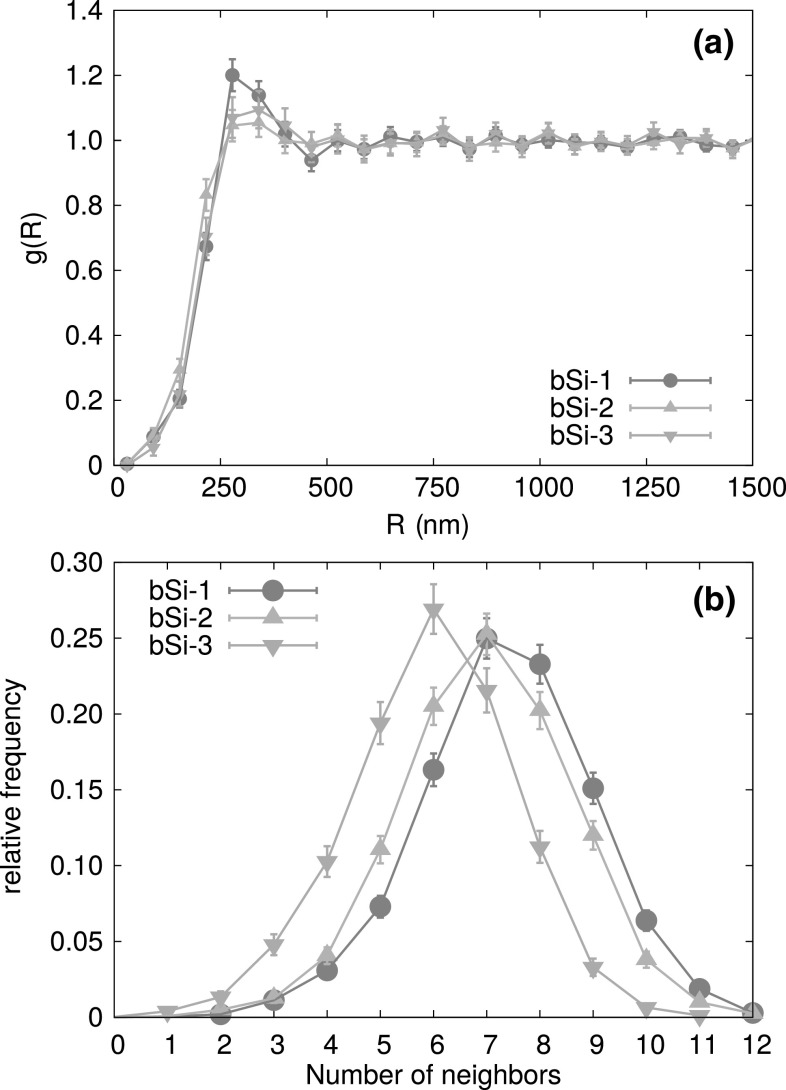
Analysis for nanopillar tip distributions. **a** Pair-correlation function (Eq. 1) of nanopillar tip positions as a function of distance between pillars for bSi-1, bSi-2, and bSi-3 substrate. **b** The distributions of numbers of neighbors found around each pillar within the first peak of the pair-correlation function with a distance less than 500 nm

In order to analyze the spatial relationship between pillars, we aim to discriminate the local pillar structure from their correlations on larger scales. We therefore identified pillar tip positions by neural network based recognition. The pair-correlation function (Eq. 1) for tip positions was determined for all types of the surfaces and presented in Fig. 3a. For small distances, from a pillar tip we observed a vanishing probability to find another pillar tip indicating that each pillar exposed an excluded area for other pillars. It was assumed that the excluded area was controlled by the scale of the pillar length and might have been influenced by the pillar bending stiffness. As can be observed from the SEM micrographs, pillars that were closer than a characteristic distance tended to lean toward each other and stand as a pillar bundle. Our detection method typically identified such pillar bundles as single pillar tip objects. With increasing distance, a first peak was visible at 306 ± 30 nm throughout all samples. The corresponding wave number (*q*) 0.020 ± 0.002 (nm^−1^) is consistent with the halo of maximum intensity in Fourier spectra as shown in Fig. S1B. We emphasize that neither Fig. 2b nor Fig. 3a show evidence for qualitative or structural differences in the top view of pillars that go beyond subtle variations of details such as the shape and height of the first peak in their pair-correlation (Fig. 3a). The overall surface density of pillars, nevertheless, controls the number of neighboring pillars seen in the vicinity of each pillar within the first peak of the pair-correlation. Figure 3b illustrates how the distribution of the number of neighbors gets shifted toward larger values with increasing density (compare Table 1).

### Bactericidal Activity

**Fig. 4:**
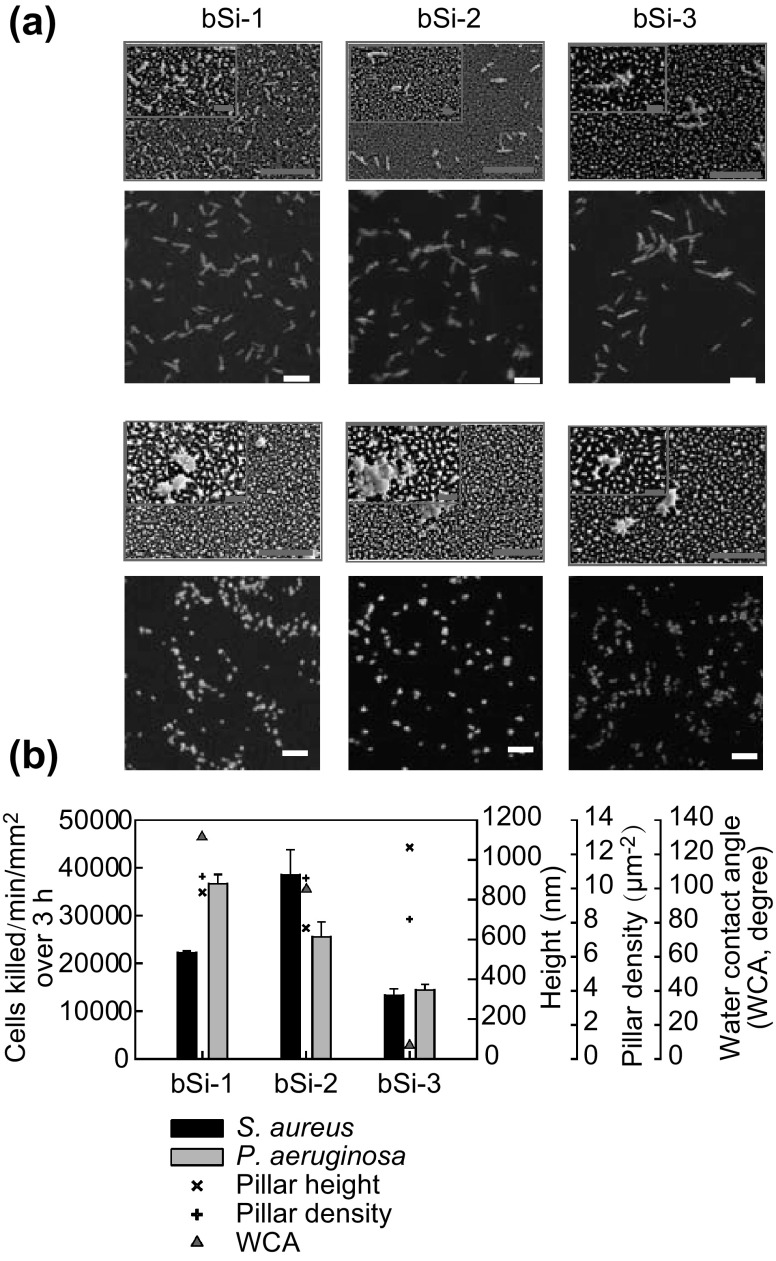
Bactericidal efficiency of the black silicon surfaces. **a** SEM images of *P. aeruginosa*, *S. aureus* cells, which appeared to be disrupted through interaction with bSi surfaces, scale bars are 1 µm. CLSM images showing the proportion of live and dead cells, live cells stained with SYTO^®^ 9 (green) and non-viable cells stained with propidium iodide (red). Scale bar is 10 µm. **b** The correlation between bactericidal efficiencies (evaluated over a period of 3 h by using a standard plate count method) and surface characteristics (topological parameters and water contact angles). (Color figure online)

Analysis of the SEM micrographs revealed the physical damage of the cells of the *P. aeruginosa* ATCC 9027 and *S. aureus* 65.8^T^ strains when attached to the bSi surfaces, although the extent of damage was a function of the type of surface (Figs. 4, S4 and Table S2). The proportion of live and dead cells, as inferred from the analysis of the CLSM images, are shown in Fig. 4a. The analysis of the results of bSi surfaces bactericidal efficiency toward *P. aeruginosa* and *S. aureus* over 3-hour incubation in nutrient-poor conditions revealed that while bSi-1 surfaces were able to effectively eliminate *P. aeruginosa* cells, *S. aureus* cells were less affected. However, bSi-2 was more efficient in elimination of *S. aureus* cells over the same period of time (3.9 × 10^4^ cells killed/min/cm^2^). Over 18-h incubation in nutrient-rich conditions, the highest proportion of *P. aeruginosa* dead cells (approximately 93%) was detected on bSi-1 surfaces, which possessed approximately 11 tips of 800 nm height per µm^2^, while the highest proportion of *S. aureus* dead cells (approximately 92%) was found on the surfaces of the bSi-2, which possessed approximately 10 tips of 600 nm height per µm^2^. In general, bSi-1 and bSi-2 were found to exhibit higher proportions of dead cell than bSi-3, the latter surface possessed the tallest pillars (up to 1000 nm) with less-dense nanopillared pattern (Table 1, Fig. 4). Overall the degree of *P. aeruginosa* and *S. aureus* dead cells on these bSi surfaces were found to be comparable, however, slightly lower than those reported in the previous study [1].

## Conclusions

The results of this work provide evidence that despite bSi substrata having a nanoarchitecture with visual similarity, including undistinguishable by neural network and FFT analysis top views, the bactericidal efficiency of such substrata can vary. The three types of bSi surfaces investigated here were varied in particular in pillar height (652.7–1063.2 nm) and density (8–12 tips per µm^2^). It is suggested that while none of the individual surface nanotopographic parameters could be directly correlated with the variations in bactericidal activity, the highest bactericidal efficiency may be achieved through the combination of different parameters. Less bactericidal bSi surfaces were found to contain nanopillars of heights reaching 1000 nm that were not always well-separated, lower pillar density (8 tips per µm^2^) and aspect ratios of 8.8. The exact relationship between the nanopattern parameters and the bactericidal properties of the surface warrant a more rigorous investigation.

## Electronic supplementary material

Below is the link to the electronic supplementary material.

Supplementary material 1 (DOCX 1155 kb)
