# Supplementary material for: Subtle Variations in Surface Properties of Black Silicon Surfaces Influence the Degree of Bactericidal Efficiency
Source: Nanomicro Lett. 2018 Feb 6;10(2):36. doi: 10.1007/s40820-017-0186-9 (PMC6199077; doi:10.1007/s40820-017-0186-9)
Supplement: Supplementary file 1 — Supplementary material 1 (DOCX 1155 kb) [file 40820_2017_186_MOESM1_ESM.docx]

Supplementary Data

Subtle Variations in Surface Properties of Black Silicon Surfaces Influence the Degree of Bactericidal Efficiency

Chris M. Bhadra^1^, Marco Werner^2^, Vladimir Baulin^2^, Vi Khanh Truong^1^, Mohammad Al Kobaisi^1^, Song Ha Nguyen^1^, Armandas Balcytis^1,3^, Saulius Juodkazis^1,3^, James Y. Wang^1^, David E. Mainwaring^1^, Russell J. Crawford^4^, Elena P. Ivanova^1*^

^1^ School of Science, Faculty of Science, Engineering and Technology, Swinburne University of Technology, Hawthorn, Victoria 3122, Australia

^2^ Departament d’Enginyeria Quimica, Universitat Rovira i Virgili, 26 Avenue dels Paisos Catalans, Tarragona 43007, Spain

^3^ Melbourne Center for Nanofabrication, 151 Wellington Road, Clayton, Victoria 3168, Australia

^4^ School of Science, RMIT University, Melbourne, Victoria 3001, Australia

*Corresponding author: [eivanova@swin.edu.au](mailto:eivanova@swin.edu.au)

**S1. Supplementary Materials and Methods**

**S1.1. Reactive ion etching**

Reactive ion etching (RIE) using SF_6_ and O_2_ was performed for 5 minutes to produce the nanopillars on the surface of silicon wafers (WRS Wafers) using an Oxford PlasmaLab 100 ICP380 instrument. The nanopillars were fabricated with a high degree of precision in terms of accuracy in size and position, allowing for a systematic study of the surface topology [1, 2]. *p*-Type boron-doped 100 mm diameter commercial Si wafers with a specific resistivity of 10-20 Ω cm^-1^, <100> oriented surface and 525 ± 25 µm thickness (Atecom Ltd, Taiwan) were used as the substrata for the fabrication of the bSi. RIE processing was performed in the mixed mode, with etching and passivation occurring simultaneously under the following conditions: SF_6_ gas flow rate of 65 sccm, O_2_ gas flow rate of 44 sccm, a pressure of 35 mTorr, 100 W RIE power, 20 °C electrode temperature and 10 Torr He backside cooling pressure. Three fabricated bSi surfaces, designated bSi-1, bSi-2 and bSi-3, were investigated in this study for their surface characteristics and bactericidal efficiency.

**S1.2. X-ray photoelectron spectrometry**

X-ray photoelectron spectroscopy (XPS) was performed using a Kratos Axis Ultra DLD X-ray photoelectron spectrometer (Kratos Analytical Ltd., UK) equipped with a monochromatic X-ray source (Al Kα, *hυ* = 1486.6 eV). The spectrometer energy scale was calibrated using the Au 4f7/2 photoelectron peak at binding energy (BE) of 83.98 eV. Samples were flooded with low-energy electrons during the analysis to counteract surface charging. The hydrocarbon component of the C 1s peak (binding energy 285.0 eV) was used as a reference for charge correction. The elements present on the surfaces were determined from survey spectra in the range 0 - 1400 eV at an interval and pass energy of 1 eV and 160 eV, respectively. The relative atomic concentration of the elements detected by XPS was quantified on the basis of the peak area in the survey spectra, using the sensitivity factors appropriate for the Kratos instrument. High resolution scans were performed across each of the C 1s, O 1s, F 1s and Si 2s peaks.

**S1.3. Cell viability analysis**

To quantify the bactericidal efficiency of bSi surfaces, two bacterial strains were incubated on each type of the surfaces for 3 hours. Viability assays were performed using a standard plate count assay [3]. *P. aeruginosa* and *S. aureus* cells were suspended in 5 mL of phosphate buffered saline (PBS) and adjusted to OD_600_ = 0.1. Re-suspended cells were diluted 1:10; then incubated in 3.5 cm diameter wells in triplicate with each well containing a 1 cm^2^ area of bSi sample. Un-modified silicon wafers were used as the control surfaces. The cell suspensions were then sampled (100 µL) at discrete time intervals over 3 h, serially diluted 1:10, and a 30 µL volume was then spread on three nutrient agar plates. The resulting colonies were then counted, and the number of colony forming units per mL was calculated. At least three independent experiments were performed. The number of colony forming units was regarded as being equivalent to the number of live cells in suspension [3]. The bactericidal efficiency was estimated as the number of inactivated cells per square centimeter of surface, per minute of incubation time, relative to the control surfaces.

**S2. Supplementary Results and Discussions**

In the previous work [1], the fabricated black silicon (bSi) exhibited tip clusters ranging from 20 – 80 nm and cluster spacing ranging from 200 to 1800nm. These surfaces have nanopillars with average height of ~500 nm. The elemental composition of the bSi surfaces was studied using X-ray photoelectron spectroscopy (XPS). A correlation between the silicon surface oxidation (Si/SiO_2_) and surface wettability could be observed (Fig. S1). The XPS data has been used to compare the atomic fractions of metallic Si, F, O, C and the Si/SiO_2_ ratios with the respective contact angles of the black silicon surfaces (Table S1). Total Si content was found to increase with decreasing wettability. The bSi-1 and bSi-2 substrates, with a contact angle of 130° and 100°, respectively, were found to have ~27% Si present on the surfaces, whereas ~35% Si was found on the bSi-4 substrate, which was superhydrophilic with a contact angle of 0°. Approximately 16% SiO_2_ was present on the surface of the bSi-4 substrate, whereas ~20-22% SiO_2_ was detected on the bSi-1 and bSi-2 (hydrophobic) substrates. This is due to the reactive ion etching (RIE) process, where oxygen ions passivate the silicon base substrate, effectively oxidizing the surface, forming SiO_2_ [4].

**Fig. S1.** (A) Correlation between surface chemistry and wettability. The atomic ratio of Si/SiO_2_ as detected by XPS; (B) The average fast Fourier transform (FFT) radial profiles of the corresponding FFT images in Fig. 2B.

High resolution scans were performed across the C 1s, O 1s, F 1s and Si 2s peaks (data not shown). The total Si elemental ratio varied between 50 to 56%, which contains Si and SiO_2_, the ratio of these two forms of silicon (Si/SiO_2_) in the surface varying between 1.2 and 2.2 (Table S1).

**Table S1.** Elemental XPS analysis of the bSi surfaces.

| Elemental atomic fractions (%) | bSi-1 | bSi-2 | bSi-3 |
| --- | --- | --- | --- |
| Si 2p | 49.8 | 50.9 | 56.3 |
| O 1s | 41.3 | 42.9 | 38.6 |
| F 1s | 0.3 | 0.6 | 0.4 |
| C 1s | 8.6 | 5.5 | 4.7 |

The elemental analysis revealed that the bSi-3 substrate was dominantly Si, with the Si composition being more than twice that if the SiO_2_. bSi-3 contained the highest level of elemental Si of all the bSi samples. As the technique demands, RIE uses chemically reactive electromagnetically generated plasma to remove material deposited on wafers under high vacuum. High-energy ions from the plasma attack the wafer surface and react with it. The probability distribution of the etching particles has been simulated using a mathematical combination of quantum mechanics, sheath dynamics and diffusion theory. This three-step model shows a probability distribution model which can predict the formation of nanostructures based on the etch rates, sheath potential to control and predict the fabrication process.

Analysis of the AFM images (Fig. S2) re-confirmed that nano-pillars varied from approximately 600 nm to 1000 nm in height, with diameters measured near the top of the nano-pillars being approximately 100 ± 10 nm and with an inter-pillar spacing of approximately 150 ± 30 nm (Table 1). The corresponding AFM line profiles of the 2D scans indicated the presence of some differences in the morphology of the pillars for each bSi substrate type.

**Fig. S2**. Surface topography of the bSi surfaces. Typical two-dimensional AFM (2.5 µm × 2.5 µm scanning areas) scans, together with the corresponding cross-sectional profiles of the black silicon surfaces. Scale bar is 2 µm.

The bearing ratio of each bSi surface type was determined from the statistical AFM data, since the protrusion curvatures were present in both the x, y and z (height) planes. The variation in the bearing ratio was dependent on the height of the nano-pillars, as shown in Fig. S3 and S4. The bearing curve reflected the surface area at a specific depth with respect to the entire area being analyzed. For all samples shown in Fig. S4, at a height of about 200 nm the bearing curve started to increase in the bearing area fraction, and approaches a constant bearing area level within several 100 nm. The increase indicated the volume of everything present above the surface, which is the volume of the pillars. It appeared that the bSi-1 and bSi-2 samples reached a constant bearing area level at approximately 500 nm, while the bSi-3 sample reached a constant bearing area level at approximately 800 nm, highlighting the nano-pillar height present on each surface.

**Fig. S3.** AFM topology analysis showing the bearing ratio as a function of the height of the nano-pillars on the respective black silicon surfaces. The bearing ratios have been obtained based on whole area scan images. The same images have been used to extract the line profiles (Supporting Data).

The morphology of the *Staphylococcus aureus* and *Pseudomonas aeruginosa* cells on the control silicon surfaces are presented in Fig. S5. It can be seen that the cells retained their usual shape and size when attached onto the control surface, without any apparent signs of damage or disruption.


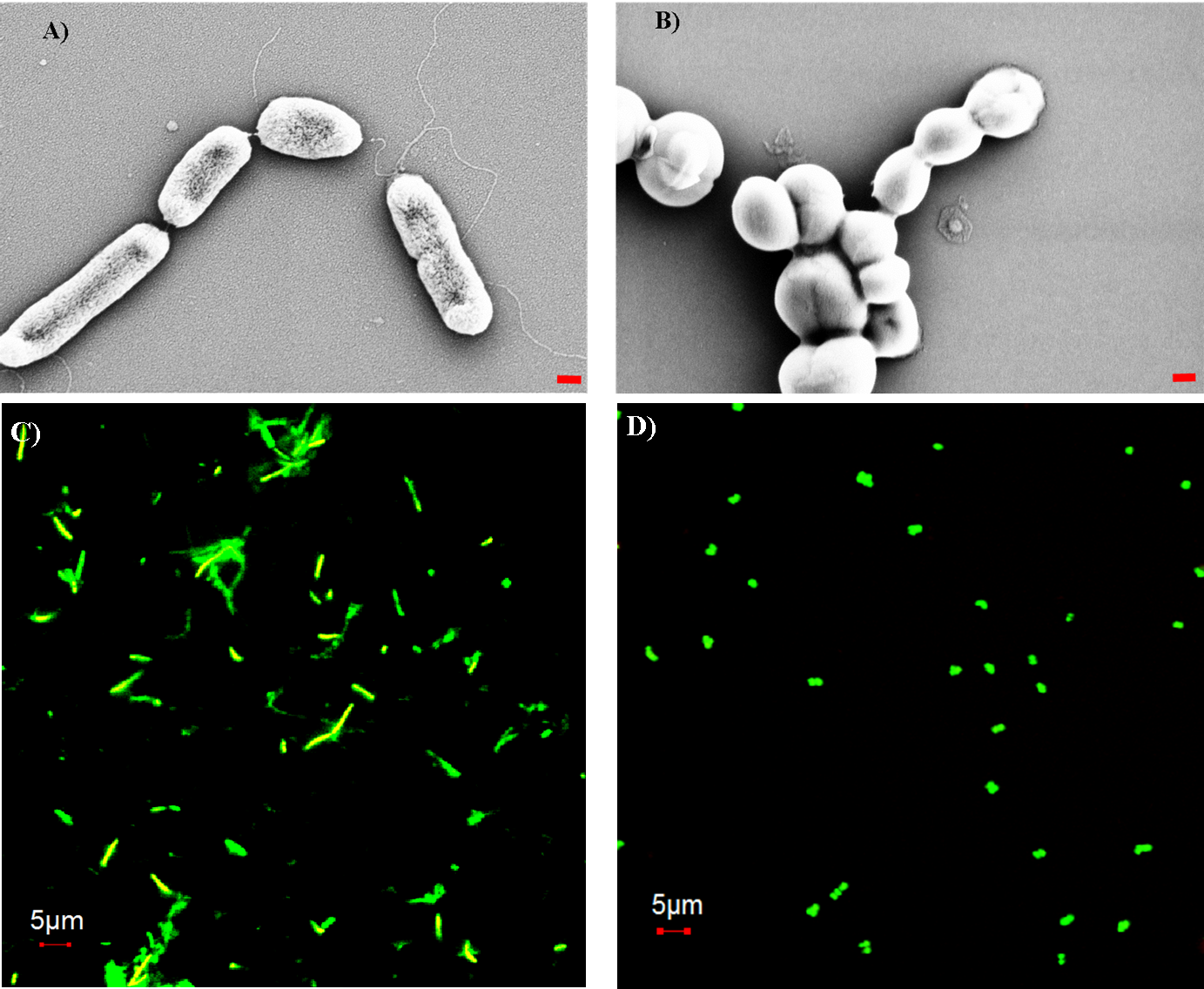


**Fig. S4. Cell morphology on control surfaces.** Typical SEM and CLSM images of intact, healthy cells of (A, C) *P. aeruginosa* and (B, D) *S. aureus* on the control Si surfaces respectively. Scale bar of SEM images: 200 nm.

**Table S2.** Bactericidal efficiency and proportion of non-viable cells of *P. aeruginosa* and *S. aureus* on bSi surfaces over two incubation conditions.

| Bacteria | Incubation conditions | bSi-1 | bSi-2 | bSi-3 |
| --- | --- | --- | --- | --- |
|  |  | Bactericidal efficiency (×10^4^ cells killed/min/cm^2^) | | |
| *P. aeruginosa* | 10 mM PBS (pH 7.4)  OD_600 nm_ = 0.1  PBS (10 mM, pH 7.4)  Dark conditions  25 °C  3 hour incubation | 3.7 ± 0.2 | 2.6 ± 0.3 | 1.4 ± 0.1 |
| *S. aureus* |  | 2.2 ± 0.1 | 3.9 ± 0.5 | 1.3 ± 0.2 |
|  |  | Proportion of non-viable cells (%) | | |
| *P. aeruginosa* | Rich nutrient conditions  OD_600 nm_ = 0.1  Nutrient broth (Difco)  Dark conditions  25 °C  18 hour incubation | 83.7 ± 8.0 | 93.9 ± 5.5 | 85.9 ± 8.7 |
| *S. aureus* |  | 92.3 ± 3.1 | 78.7 ± 7.3 | 73.5 ± 10.8 |

1. E.P. Ivanova, J. Hasan, H.K. Webb, G. Gervinskas, S. Juodkazis, V.K. Truong, A.H. Wu, R.N. Lamb, V.A. Baulin, G.S. Watson, Bactericidal activity of black silicon. Nat. Commun. **4**(2838 (2013). doi: 10.1038/ncomms3838.

2. H. Jansen, M. De Boer, R. Legtenberg, M. Elwenspoek, The black silicon method: A universal method for determining the parameter setting of a fluorine-based reactive ion etcher in deep silicon trench etching with profile control. J. Micromech. Microeng. **5**(2), 115-20 (1995). doi: 10.1088/0960-1317/5/2/015.

3. J.R. Postgate. Chapter XVIII Viable Counts and Viability. Methods in Microbiology1969. p. 611-28.

4. X. Liu, P.R. Coxon, M. Peters, B. Hoex, J.M. Cole, D.J. Fray, Black silicon: Fabrication methods, properties and solar energy applications. Energy Environ. Sci. **7**(10), 3223-63 (2014). doi: 10.1039/c4ee01152j.
